# Highly Selective Electrocatalytic CuEDTA Reduction by MoS_2_ Nanosheets for Efficient Pollutant Removal and Simultaneous Electric Power Output

**DOI:** 10.1007/s40820-023-01166-7

**Published:** 2023-08-09

**Authors:** Hehe Qin, Xinru Liu, Xiangyun Liu, Hongying Zhao, Shun Mao

**Affiliations:** 1 College of Environmental Science and Engineering, Biomedical Multidisciplinary Innovation Research Institute, Shanghai East Hospital, State Key Laboratory of Pollution Control and Resource Reuse, Tongji University, 1239 Siping Road, Shanghai, 200092 People’s Republic of China; 2 Shanghai Institute of Pollution Control and Ecological Security, Shanghai, 200092 People’s Republic of China; 3 Shanghai Key Lab of Chemical Assessment and Sustainability, School of Chemical Science and Engineering, Tongji University, 1239 Siping Road, Shanghai, 200092 People’s Republic of China

**Keywords:** Electrocatalytic reduction, CuEDTA removal, MoS_2_ nanosheet, CuEDTA/Zn battery, Faraday efficiency

## Abstract

Electrocatalytic reduction of ethylenediamine tetraacetic acid copper (CuEDTA), a typical refractory heavy metal complexation pollutant, is an environmental benign method that operates at mild condition. Unfortunately, the selective reduction of CuEDTA is still a big challenge in cathodic process. In this work, we report a MoS_2_ nanosheet/graphite felt (GF) cathode, which achieves an average Faraday efficiency of 29.6% and specific removal rate (SRR) of 0.042 mol/cm^2^/h for CuEDTA at − 0.65 V vs SCE (saturated calomel electrode), both of which are much higher than those of the commonly reported electrooxidation technology-based removal systems. Moreover, a proof-of-concept CuEDTA/Zn battery with Zn anode and MoS_2_/GF cathode is demonstrated, which has bifunctions of simultaneous CuEDTA removal and energy output. This is one of the pioneer studies on the electrocatalytic reduction of heavy metal complex and CuEDTA/Zn battery, which brings new insights in developing efficient electrocatalytic reduction system for pollution control and energy output.

**Supplementary Information:**

The online version contains supplementary material available at 10.1007/s40820-023-01166-7.

## Highlights

- Highly efficient CuEDTA removal by an electrolyzer with MoS_2_ nanosheet cathode.
- Higher removal rate and Faraday efficiency compared with other widely reported electrocatalytic technologies.
- CuEDTA/Zn primary battery is constructed for the first time to realize CuEDTA removal and synchronous power generation.

**Supplementary Information:**

The online version contains supplementary material available at 10.1007/s40820-023-01166-7.

## Graphical abstract

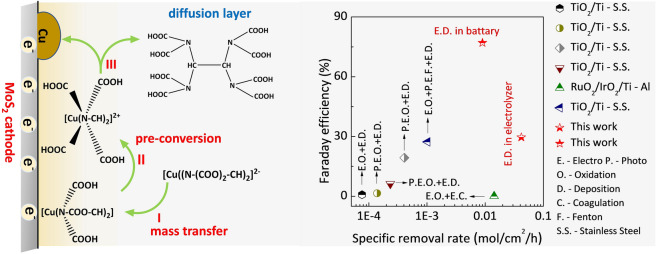

## Introduction

Ethylenediamine tetraacetic acid copper (CuEDTA), one of the typical refractory and toxic heavy metal complexation pollutants, is massively discharged from industrial processes including electroplating and metallurgy [1]. The extremely stable structure and highly soluble property of CuEDTA over a wide pH range make it quite challenge to apply the traditional precipitation treatment method for this heavy metal complexation [2]. On the other hand, as a valuable metal, the recovery of Cu from CuEDTA wastewater is important for both resource utilization and pollution control [3, 4]. To realize the treatment and recovery of the complexes, a common strategy is decomplexation by oxidation coupled with the deposition of metal [2, 3, 5–13]. However, obstacles of high energy consumption and low removal or recovery rate/efficiency prevent the practical applications of this method. For instance, Wang et. al. reported the oxidative decomplexation of CuEDTA by HCO_3_^−^/H_2_O_2_-based advanced oxidation technique, which required ~ 2000 times of H_2_O_2_ dosage to achieve 40–92% decomplexation and 74.8% total organic carbon (TOC) removal of 0.2 mM CuEDTA in 60 min [14]. Therefore, more efficient technology for metal–organic complex removal is highly needed.

Electrochemical reduction is an electrode interface reaction process, in which the reactant gains electrons directly at the electrode, and the reaction proceeds at a certain rate when an appropriate overpotential is applied [15, 16]. Cathodic reduction has unique advantage in CuEDTA removal since Cu is directly precipitated by cathodic reduction through a two-electron process of the central Cu^2+^ without ligand degradation [17]. Then, the de-complexed EDTA can either be recycled or removed using less costly biological treatment techniques, which shows lower energy consumption than that of the anodic decomplexation/precipitation process [17, 18]. Direct electrochemical reduction of CuEDTA has been reported in previous studies [17–21]. However, these studies mainly focus on the operation parameter optimizations in the reactor including solution pH, electrolyte, current density, flow rate, etc. Although high Cu recovery efficiency can be achieved in these systems, the current efficiency is relatively low (25%) even with CuEDTA concentrations up to 10 mM [17, 21], which greatly limits the practical applications of this technology due to the unfordable energy consumption. Another important issue in electrochemical reduction of CuEDTA is that the hydrogen evolution reaction (HER) at the cathode is ignored in most cases, which is a competitive reaction for CuEDTA reduction reaction (CuRR) in cathodic reaction. To address these challenges, highly selective and efficient cathode for CuRR is needed to realize the practical applications of this technology.

2D MoS_2_ nanosheet (NS) has exposed edges and basal plane, which shows unique surface chemistry properties compared with its bulk form [22, 23]. MoS_2_ nanosheets have been reported in electrocatalytic reactions including N_2_ or NO reduction reaction [24–26], CO_2_ reduction reaction [27], O_2_ reduction reaction [28, 29], etc. In one study, Zhang et al. reported the electrochemical reduction of NO on MoS_2_ (1 0 1) crystalline surface. They showed that MoS_2_ was more conducive to NO adsorption, and the N–O bond of adsorbed NO was elongated, which benefited the subsequent electroreduction process [24]. Motivated by these studies, we speculate that MoS_2_ nanosheet may have promising catalytic activity in cathodic reduction of CuEDTA.

In this work, we report a MoS_2_ nanosheet-based electrode with abundant exposed edges for selective CuEDTA reduction. This cathode achieves high catalytic performance for CuRR, which is superior to commonly used cathodes in electrocatalytic systems including Pt/C, Cu, and carbon black [30]. Based on the density functional theory (DFT) modeling, it is shown that the activation energy of CuRR on the MoS_2_ surface is 0.114 eV, which is much lower than that of the graphite electrode (0.821 eV). Considering the outstanding activity of MoS_2_ nanosheet for CuRR, two types of electrochemical devices, i.e., electrolyzer and CuEDTA/Zn battery, are constructed and demonstrated by the MoS_2_/graphite felt (GF) cathode. In the electrolyzer, the MoS_2_/GF achieves an average Faraday efficiency (FE) of 29.6% and a high specific removal rate (SRR) of 0.042 mol cm^−2^ h^−1^ for CuEDTA at − 0.65 V versus SCE, both of which are higher than those of the (photo)electrooxidation technology-based removal systems [3, 31]. In the CuEDTA/Zn battery, a maximum power density of 1.05 mW cm^−2^ at 1.95 mA cm^−2^ can be delivered. The MoS_2_/GF cathode battery can achieve 75% removal of CuEDTA with a superhigh FE of 77%. To the best of our knowledge, this is the first report of CuRR coupled with battery application for the CuEDTA treatment and energy storage. The reported highly efficient and low energy consumption electrolyzer for CuEDTA removal and CuEDTA/Zn battery present new insights in the practical applications of electrochemical system for heavy metal complex treatment and energy reuse.

## Experimental

### Preparation of MoS_2_/GF Cathode

The MoS_2_/GF cathode was prepared by the hydrothermal synthesis method. In detail, 1 mmol Na_2_MoO_4_ and 5 mmol urea were dissolved in 30 mL ultrapure water. The GF (6 cm × 2 cm) was washed with dichloromethane, ethanol, and 0.1 M hydrochloric acid, successively. Then, the mixed liquor was transferred into a Teflon-lined stainless-steel autoclave. The washed GF was rolled and placed into the autoclave. After 24 h hydrothermal reaction at 200 °C, the MoS_2_ nanosheets were produced and deposited on the GF surface. The MoS_2_/GF was dried in vacuum and cut into small pieces for later use.

### CuEDTA Removal by Electrolyzer

The CuEDTA electroreduction experiment was carried out in the two-compartment reaction cell separated by the ion exchange membrane. The MoS_2_/GF was used as the cathode (size of 3 cm^2^) and Pt plate was used as the anode. The CuEDTA (1 mM) and Na_2_SO_4_ (0.5 M) was used as the electrolyte for the cathode and anode compartment, respectively, and the solution volume was 30 mL for each compartment. A polymer electrolyte membrane (PEM) was used to separate the cathode and anode. During the reaction, at a predetermined sampling time, 0.25 mL reaction solution was collected and diluted with 0.25 mL H_2_O for real-time CuEDTA concentration determination or 0.25 mL of 1 mM CuSO_4_ solution for total CuEDTA concentration determination by high-performance liquid chromatography test (HPLC, Agilent 1260, USA). The real-time concentration of EDTA was obtained by subtracting the real-time CuEDTA from the total concentration. The test conditions of HPLC were as follows: the test wavelength was 225 nm, the column was C_18_ (4.6 × 250 mm), the volume ratio of 10 mM H_3_PO_4_ to acetonitrile water was 92:8, and the flow rate was 1 mL min^−1^. The standard curve of CuEDTA concentration determined by HPLC is shown Fig. S1. The electrode regeneration was achieved by soaking the Cu-deposited MoS_2_/GF in saturated ethylenediamine tetraacetic acid for 24 h. Tap water and surface water (filtered by 0.44 µm water phase needle filter, from Tongji University campus) were used to study the impact of coexist chemicals in real water samples on the electroreduction efficiency of CuEDTA in our system.

### CuEDTA/Zn Battery Test

The CuEDTA/Zn battery was constructed in the two-compartment reaction cell separated by the bipolar ion exchange membrane (BIEM). The MoS_2_/GF was used as the cathode (size of 3 cm^2^) and Zn plate was used as the anode. The Na_2_SO_4_ (0.5 M) and KOH (1 M) was used as the electrolyte for the cathode and anode compartment, respectively, and the solution volume was 30 mL for each compartment. The output characteristics of the battery were measured by chronopotentiometry with current ramp method, and the current sweep speed was 0.1 mA s^−1^. The CuEDTA concentration was measured with the same method using HPLC. Chronopotentiometry was used to measure the potential change under constant current discharge condition. In both electrolyzer and battery cells, Ar was continuously pumped to prevent interference of oxygen and carbon dioxide in the air. Other experimental details of material characterizations and electrochemical tests are shown in the Supporting Information (Text S2–S3).

## Results and Discussion

### Reactor Design and Electrode Characterizations

**Fig. 1:**
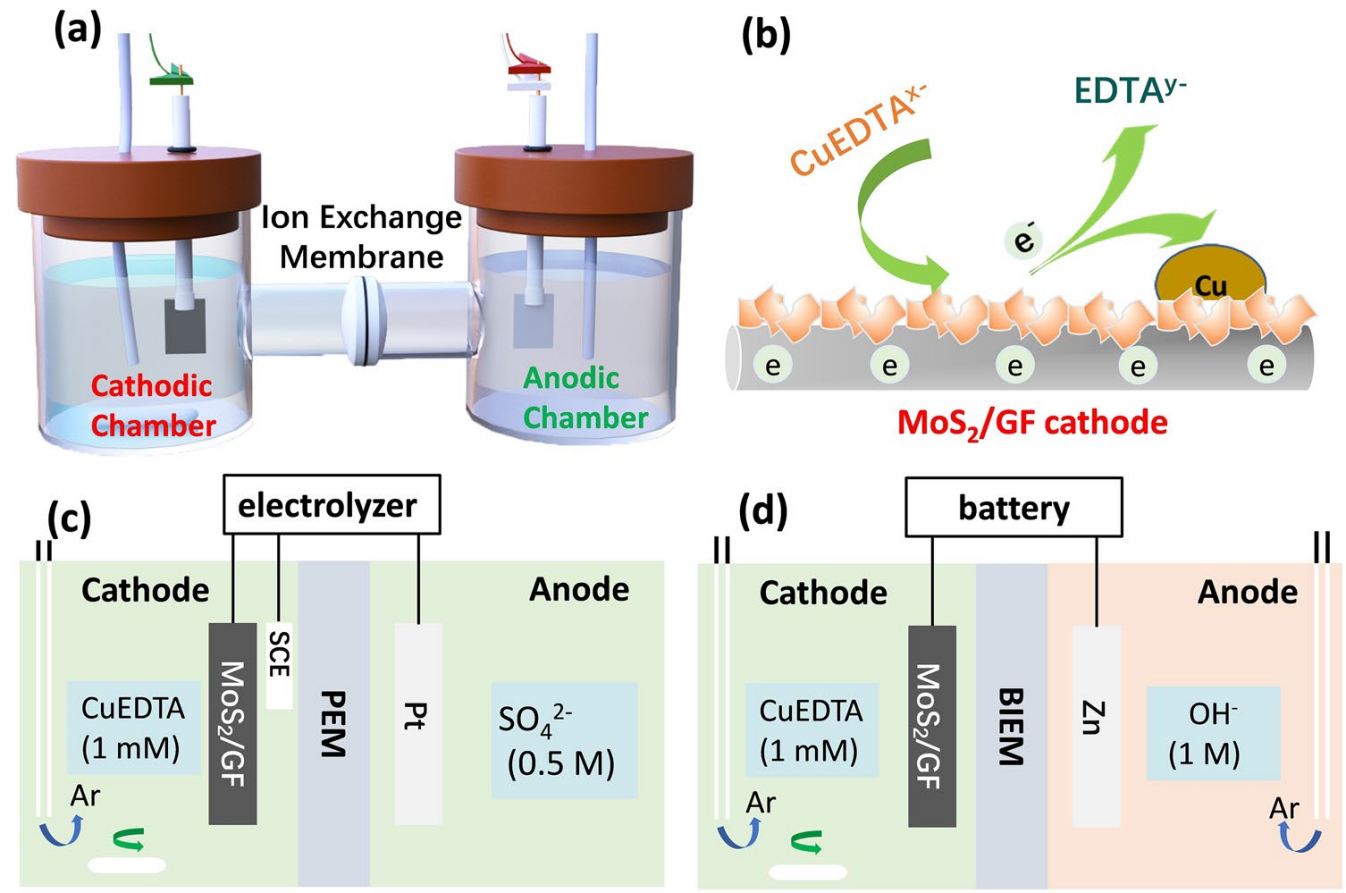
**a** Schematic diagram of the reaction system. **b** CuEDTA reduction reaction on the MoS_2_/GF cathode. **c, d** Schematic diagrams of the electrolyzer and CuEDTA/Zn battery

Figure 1a shows the experimental device used in this study, which is a two-compartment reaction cell separated by an ion exchange membrane. The core reaction of the reactor is the CuRR on the cathode, which occurs in both electrolyzer and primary battery. With the MoS_2_/GF cathode, the CuEDTA decomplexation and reduction proceed to produce copper deposition on the cathode surface (Fig. 1b). In the electrolytic cell system, the CuEDTA is removed and Cu is recovered by the applied voltage; while the oxygen evolution occurs at the counter electrode (Fig. 1c). In the CuEDTA/Zn battery, the redox reaction between Zn and CuEDTA occurs spontaneously and generates electrical energy (Fig. 1d). In order to eliminate the interference of oxygen and other reactive gases, the devices were operated under the protection of argon.

**Fig. 2:**
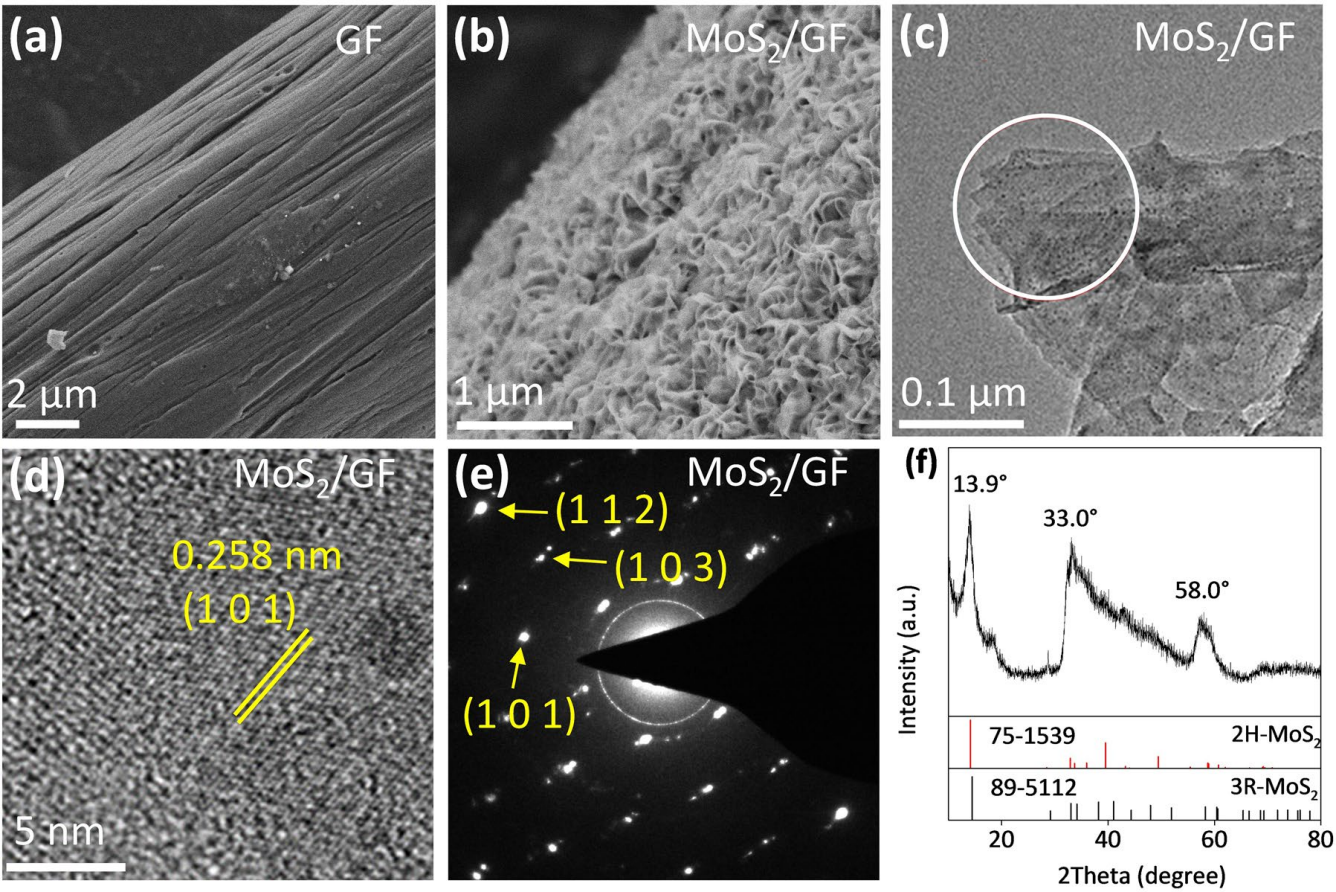
SEM images of **a** bare GF and **b** MoS_2_/GF. **c** TEM and **d** HRTEM images of MoS_2_ nanosheet. **e** The corresponding SEAD patterns of MoS_2_ in the circle location of **c**. **f** XRD patterns of MoS_2_

The prepared MoS_2_ nanosheets with exposed edges on the GF electrode are first characterized. As shown in the scanning electron microscope (SEM) image, the GF electrode has a smooth surface (Fig. 2a). After the in-situ synthesis of MoS_2_ nanosheets, the GF is fully covered by a dense MoS_2_ layer (Fig. 2b). The nanosheet feature is evidenced by the transmission electron microscope (TEM) image shown in Fig. 2c, which indicates that the MoS_2_ nanosheets have a vertically-aligned structure with large number of exposed edges. The high-resolution TEM (HRTEM) image (Fig. 2d) and selected area electron diffraction (SAED) patterns (Fig. 2e) confirm the crystalline structure of MoS_2_. The 0.258 nm lattice fringe in the HRTEM image can be indexed to the (1 0 1) crystal plane of hexagonal MoS_2_ crystalline.

The X-ray diffraction (XRD) patterns in Fig. 2f show diffraction peaks at 13.9°, 33.0°, and 58.0°, which may be attributed to the diffraction peaks of polytype 2H-MoS_2_ or 3R-MoS_2_ [32, 33]. The Raman spectroscopy was further obtained to investigate the structure of the prepared MoS_2_ nanosheet (Fig. S2). The MoS_2_ nanosheet exhibits two vibration bands at 385 cm^–1^ (E_2g_) and 405 cm^–1^ (A_1g_), which demonstrates that the 2H-MoS_2_ phase is the major constituent [34]. The energy dispersive spectroscopy (EDS) spectrum of the prepared MoS_2_ nanosheet is shown in Fig. S3. In addition to Mo and S, there is also a certain amount of C, which may be attributed to the carbon in the organic thiourea from which MoS_2_ is synthesized. The atomic ratio of S to Mo is measured as 1.95, further confirming the successful preparation of MoS_2_.

The surface chemical states of MoS_2_ were characterized by X-ray photoelectron spectroscopy (XPS). As shown in Fig. S4, the peaks at 236.0, 232.8, and 229.3 eV can be attributed to Mo(VI) 3*d*_3/2_ of MoS_2_ surface plasmon, Mo(IV) 3*d*_3/2_ and Mo(IV) 3*d*_5/2_ of MoS_2_, respectively. The extra peak at ∼ 226.3 eV in Mo 3*d* XPS is referred as S 2*s*. The peak at 162.3 eV corresponds to Mo^+^ state in S 2*p*_3/2_, and the peaks at 163.3 and 169 eV are Mo^2+^/MoS_2_ surface plasmon state in S 2*p*_1/2_. The XPS results are consistent with the reported electronic structure of 2D MoS_2_ thin film [35].

### Electrochemical Characterizations and CuRR Mechanism

Before the device demonstration of the electrochemical CuEDTA removal, electrochemical characterizations were performed to study the electrode process of CuRR on the MoS_2_ cathode. The catalytic activity of the prepared catalyst towards CuRR in 0.5 M Na_2_SO_4_ solution was firstly evaluated by cyclic voltammetry (CV) measurements (Fig. S5). For comparison, the voltammograms of commonly used Pt/C, Cu, carbon black (CB), and glassy carbon (GC) electrodes in electrochemical reduction territory were also tested. Compared with the CV curves recorded in the Na_2_EDTA solution (red curve), the redox currents were observed for MoS_2_ electrode, while the other electrodes showed no obvious redox currents, indicating that the MoS_2_ nanosheet has superior catalytic activity for CuRR.

**Fig. 3:**
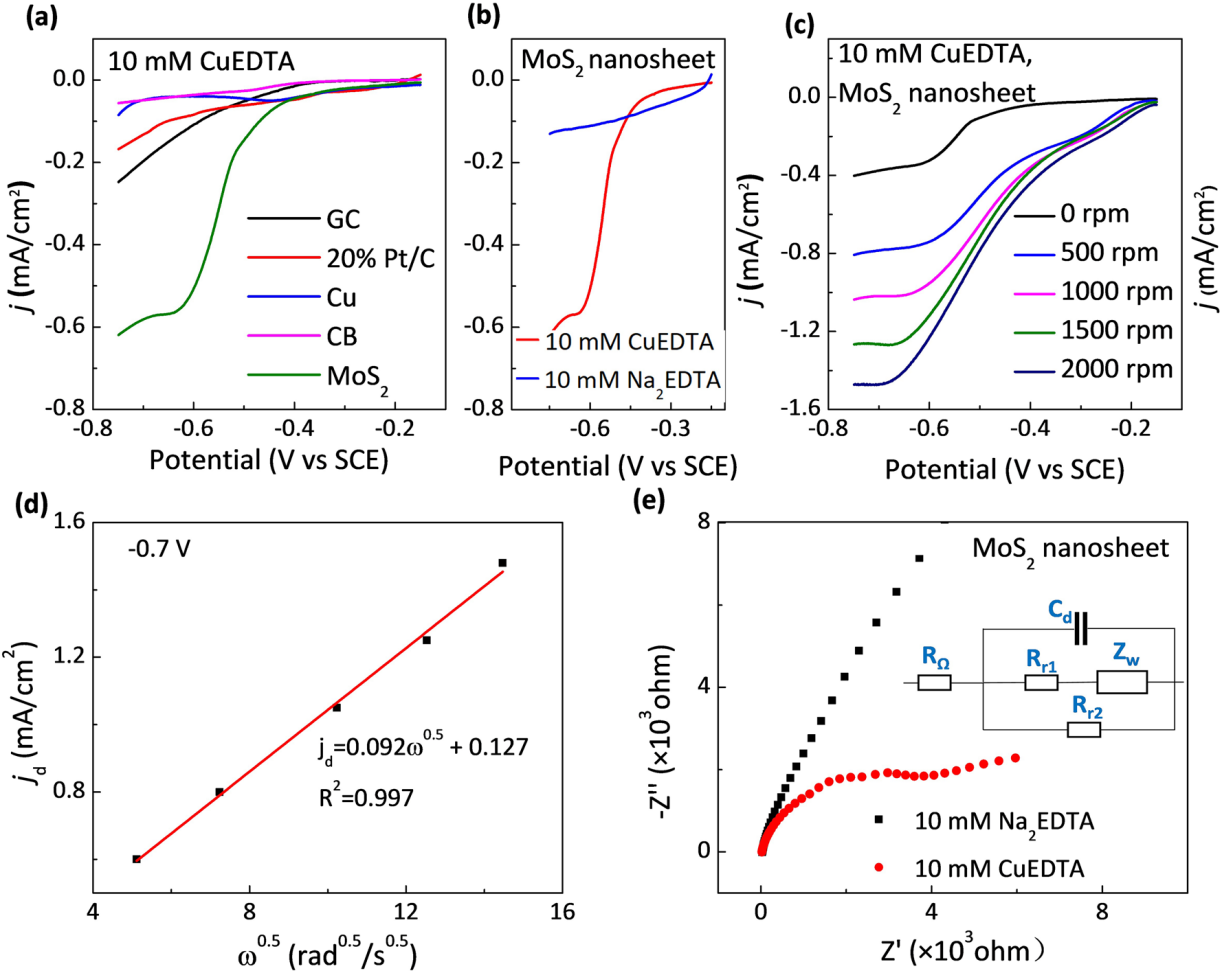
**a** LSVs of different cathodes for CuRR (10 mM CuEDTA, rotation speed of 250 rpm). **b** LSVs of the MoS_2_/GF cathode for HER (10 mM Na_2_EDTA, rotation speed of 250 rpm) and CuRR (10 mM CuEDTA, rotation speed of 250 rpm). **c** LSVs of the MoS_2_/GF cathode for CuRR at different rotation speeds. **d** $j_{{{d}}} - \omega^{1/2}$ plot at − 0.75 V versus SCE. **e** Nyquist plots of the MoS_2_ cathode for CuRR and HER at − 0.75 V versus SCE. The inset shows the equivalent circuit of the electrochemical system. *C*_d_ is the differential capacitance, *R* is the solution resistance (*R*_Ω_) or reaction resistance (*R*_r1_ and *R*_r2_), and Z_w_ is the electrochemical reaction and mass transfer complex impedance

As shown in Fig. 3a, the linear sweep voltammetry (LSV) curves further indicate that the MoS_2_ cathode has higher electrochemical activity for CuRR, and its reduction current exceeds those of the Pt/C, Cu, CB, and GC electrodes. Since the thermodynamic standard potential of CuEDTA reduction is lower than that of HER (Text S1), it is inevitable that the CuRR and HER will occur simultaneously. Thus, we further compared the HER and CuRR currents of the MoS_2_ cathode to investigate its activity and selectivity for these two competing reactions.

For the MoS_2_ electrode, Fig. 3b clearly shows that the reduction current of CuRR (10 mM CuEDTA) is greater than that of HER (10 mM Na_2_EDTA), indicating that MoS_2_ nanosheet cathode has a strong selectivity for CuRR over HER. In contrast, the other cathodes show different activities in CuRR and HER. As shown Fig. S6, the HER performance is excellent for Pt/C. However, the cathodic current is greatly inhibited in the presence of CuEDTA, which indicates that the Pt/C cathode has an intrinsic inferior activity of CuRR compared with HER. As for Cu cathode, the similar decrease in current density is found in the presence of CuEDTA, which may also be ascribed to the inherent lower catalytic activity for CuRR than HER. No difference is observed between the HER and CuRR currents on the CB cathode since the activity of CB cathode is low for both reactions. The above results indicate that MoS_2_ nanosheet has the strongest CuRR selectivity among the studied cathodes.

The limit current of CuRR on MoS_2_ cathode can be used to verify if the reaction is controlled by non-electron-transfer steps like mass transfer or pre-conversion steps. Hence, we carried out rotating disk electrode tests at different rotate speeds to reveal if the CuRR on MoS_2_ electrode was controlled by the mass transfer step. As shown in Fig. 3c, the limit current density of CuRR increases with the increased rotation speed, indicating that, in the tested potential range and rotation speed, the CuEDTA reduction reaction is controlled by the mass transfer process. Similarly, we have also carried out the rotating disk tests on the other cathodes (Fig. S6). Different from the results on MoS_2_ cathode, no difference was observed at various rotation speeds, indicating the non-mass-transfer controlled reaction current and their limited electrocatalytic reduction performances. The high catalytic activity and selectivity of the MoS_2_ cathode for CuRR can also be confirmed from the Nyquist plots (Fig. 3e), which shows smaller radius of curvature for CuRR compared with that of HER. On the other reference electrodes, there is no significant difference in the reaction resistance between HER and CuRR (Fig. S7). In addition, according to the fitting curve of the relationship between the limiting current density (*j*_d_) and the rotational angular velocity (*ω*^1/2^) in Fig. 3d, the electron transfer number is calculated to be ~ 1.9, indicating that the two-electron CuRR process is the main process in the system.

**Fig. 4:**
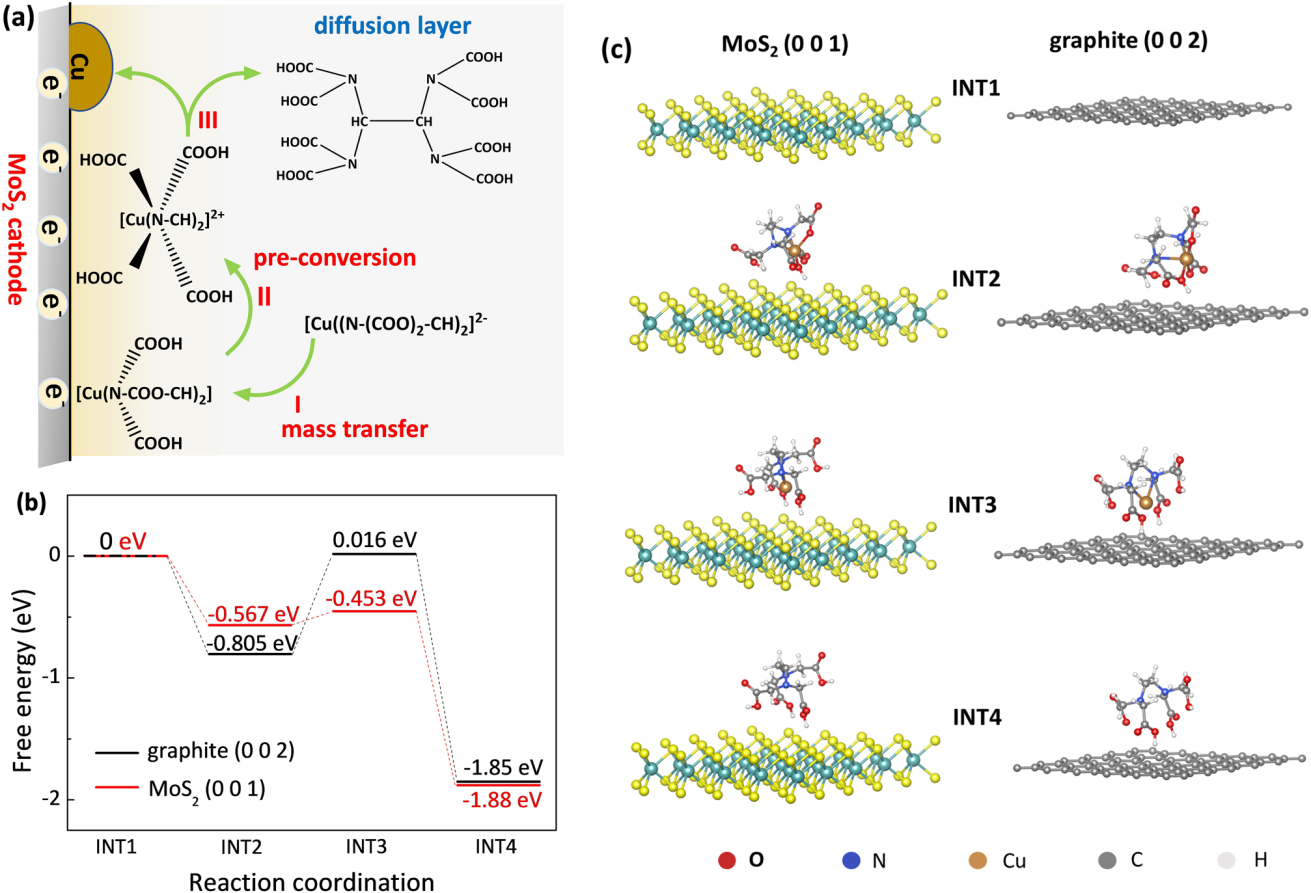
**a** Schematic diagram of electrocatalytic reduction of CuEDTA at the MoS_2_ cathode. **b** Energy states of different CuEDTA conformations on the MoS_2_ (1 0 0) and graphite (0 0 2) surfaces. **c** Modeling of the CuEDTA reduction on the MoS_2_ and graphite surface in the reaction process. INT1 to INT4 represent four reaction states of CuEDTA from 6 to 0 coordination

Figure 4a shows the schematic diagram of electrocatalytic reduction process of CuEDTA at the MoS_2_ cathode. For the electrocatalytic CuEDTA reduction, the first step is the mass transfer process of the CuEDTA to the cathode surface. Based on the above electrochemical test results, we have shown that CuRR is limited by the mass transfer on MoS_2_ cathode; while the CuRR on the other cathodes is limited by their inherently poor catalytic activity in the pre-conversion step under good mass transfer conditions (Text S5). In general, the vacant orbital of the central metal ion of the coordination compound is filled with lone pair electrons of the ligands; while, the existence of the vacant orbital is necessary to achieve the reduction of the central metal ion, which is the pre-conversion process. The pre-conversion process largely determines the overall efficiency of the CuRR, in which the decomplexation reaction of CuEDTA on the cathode can be expressed by the following equations:

1 $${\text{CuEDTA}} \left( 6 \right) \to {\text{CuEDTA }}\left( 4 \right)$$

2 $${\text{CuEDTA}} \left( 4 \right) \to {\text{CuEDTA}} \left( 2 \right)$$

3 $${\text{CuEDTA}} \left( 2 \right) \to {\text{Cu}}^{2 + } + {\text{EDTA}}^{2 - }$$

where the numbers in parentheses represent the coordination numbers of Cu^2+^.

To understand how the MoS_2_ nanosheets catalyze the CuRR process, DFT calculation was utilized to investigate the multi-step decomplexation process in CuEDTA reduction (Text S6). The change of free energy of CuEDTA in the pre-conversion process on the MoS_2_ surface and graphite surface was calculated for comparison (Fig. 4c). As shown in Fig. 4b, we stipulate that the initial energy of six-coordinated Cu on the (1 0 0) crystal plane of MoS_2_ and the (0 0 2) crystal plane of graphite is 0 eV. Because of the Jahn–Teller effect, the energy that is needed for the CuEDTA to transform to the tetra-coordination state is decreased, which means that the transform of CuEDTA from 6 to 4 coordination is a spontaneous process. In contrast, the activation free energy of CuEDTA from four-coordinated to two-coordinated Cu is 0.821 eV for graphite and 0.114 eV for MoS_2_ nanosheet, respectively. This result clearly indicates that the energy required for the decomplexation of CuEDTA on the MoS_2_ surface is much lower than that on the graphite surface; thus, the speed of this step on the MoS_2_ cathode is faster than that on the carbon cathode. The DFT calculation results conform the inherent high activity of MoS_2_ nanosheet for the decomplexation of CuEDTA, which theoretically support that the MoS_2_ nanosheet cathode is suitable for electrochemical reduction removal of CuEDTA. This result also indicates that, for the electrochemical reduction removal of heavy metal complexes, the catalyst with low energy barrier for the conformation change of the complex is critical for the efficient reduction of heavy metal complexes.

### CuEDTA Removal in Electrolyzer

**Fig. 5:**
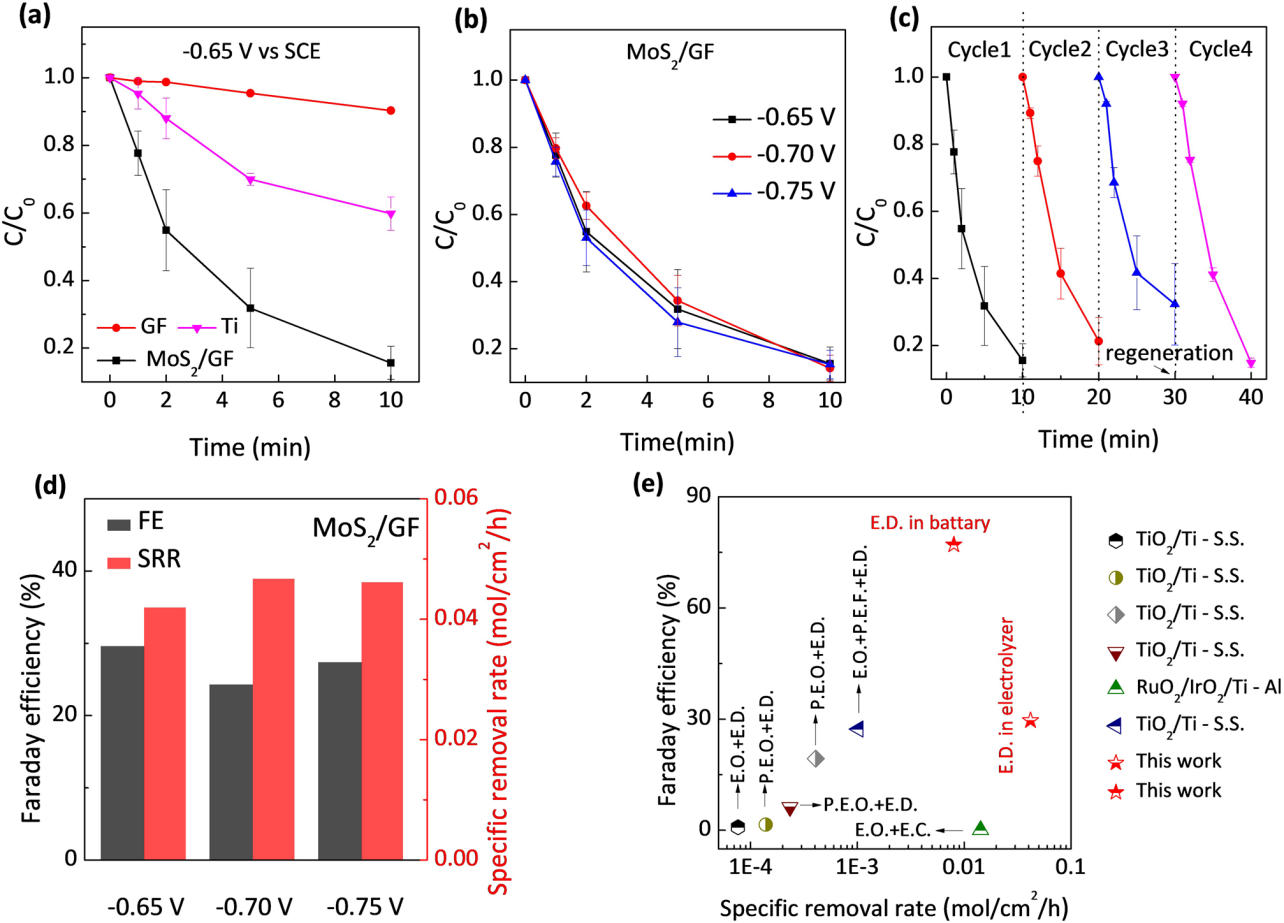
Removal performance of CuEDTA **a** by MoS_2_/GF, GF, and Ti cathodes; and **b** at different operation potentials by MoS_2_/GF cathode. **c** Reusability of the MoS_2_/GF cathode. **d** Faraday efficiency and special removal rate at different potentials with the MoS_2_/GF cathode. Working condition: 1 mM CuEDTA, 0.5 M Na_2_SO_4_, 30 mL solution, 3 cm^2^ electrode surface area. **e** Comparison of the FE and SRR of our MoS_2_/GF system with other reported electrooxidation-based CuEDTA removal systems (E. represents electro, P. represents photo, O. represents oxidation, D. represents deposition, C. represents coagulation, F. represents Fenton, and S.S represents stainless steel)

In view of the excellent activity and selectivity of MoS_2_ nanosheet for CuRR, two proof-of-concept devices were constructed to investigate the practical application prospects of CuEDTA removal, which is the electrolyzer for CuEDTA removal and the CuEDTA-Zn battery (MoS_2_/GF, CuEDTA (SO_4_^2−^)||OH^−^, Zn) for simultaneous Cu removal and energy output. The electroreduction removal of CuEDTA wastewater in the electrolyzer was first investigated. The CuEDTA can be directly removed by anodic oxidation at the oxygen evolution potential window according to previous report [36–38]. Therefore, in order to eliminate the interference of anodic electrooxidation, the two-chamber electrolyzer was used. Different cathodes including MoS_2_/GF, Ti, and GF were studied and compared in the electrolyzer. As shown in Fig. 5a, under good mass transfer condition by forced convection, CuEDTA can be removed fast with 84% removal rate in 10 min with the MoS_2_/GF cathode, which is much higher than the Ti plate (40% removal rate in 10 min) that was commonly adopted as the cathode [11, 18], and is also higher than that of the GF cathode. The high removal rate of CuEDTA by the MoS_2_/GF cathode further confirms that the MoS_2_ nanosheet has outstanding catalytic performance in CuRR.

The potentiostatic method was adopted to further study the mass-transfer controlled removal process. In the experiment, three different cathodic potentials of − 0.65, − 0.70, and − 0.75 V (vs. SCE) were applied. At these potentials, the removal rates of CuEDTA are almost the same as shown in Fig. 5b. The results match well with the electrochemical characterization results, which indicate that the mass transfer rate determines the CuRR process in the applied potential range. The current density of the cathode at three different cathodic potentials were also recorded (Fig. S8). The HER current density reaches the stable status rapidly after the transient unstable electrode process (capacitor charging current and Faraday reaction current); while the CuRR current density gradually decreases. The reason for the CuRR current drop can be understood by the following dynamic equation of mass transfer-controlled electrode process:

4 $$j = nFD\frac{{c - c_{{{s}}} }}{l}$$

where *C* is the reactant concentration in solution, *C*_s_ is reactant concentration at electrode surface, *n* is the electron transfer number, F is the Faraday constant, *D* is the diffusion coefficient of CuEDTA, *l* is the thickness of the diffusion layer. As the reactant concentration, i.e., the CuEDTA concentration, decreases, the current density decreases. When the current density of CuRR becomes zero, the remaining cathode current is attributed to the HER current. It is found that the current densities are close under the applied cathodic potentials, which matches with the CuEDTA removal rates under different cathodic potentials (Fig. 5b).

The electroreduction of CuEDTA is an unstable or unsustainable process due to the coverage of the MoS_2_/GF cathode by Cu deposition. To evaluate the durability of the cathode, the recycling test was carried out. As shown in Fig. 5c, after 3 cycles, a degradation of the cathode performance is noticed. The SEM images of the cathode surface after the reaction confirm the deposition and coverage of Cu on the cathode (Fig. S9). The Cu-deposited electrode can be refreshed in saturated ethylene diamine tetraacetic acid, and its catalytic activity for CuEDTA removal can be fully recovered as shown in the 4th cycle. The above results show that the reported MoS_2_/GF cathode has good practical application potential for CuEDTA wastewater treatment.

Furtherly, we investigated the impact of coexist chemicals in real environment water on the CuEDTA removal efficiency of our system. As shown in Fig. S10, it is found that the removal efficiencies of CuEDTA in tap water and pure water are almost the same; while the removal efficiency in surface water (100% CuEDTA removal in 10 min) is even higher. The results show that the coexist chemicals in tap water and surface water have no inhibiting effect on CuEDTA removal, demonstrating the potential of the system for practical environmental application. In addition to CuEDTA, we also measured the EDTA concentration during the electrochemical reduction process (Fig. S11). The results show that the ligand EDTA in CuEDTA remains unchanged during the electrode reaction process. The ligands alone are not toxic and can be further processed through inexpensive biological processes or recycled as industrial products. These results further demonstrate the potential of the system for practical environmental applications.

By integrating the chronoamperometric curve, the FE of CuEDTA removal at different potentials was obtained (Text S4). The specific removal rate (SRR) and FE of the MoS_2_/GF cathode at the applied potentials are close, mainly due to the mass transfer limitation and the high selectivity for CuRR of MoS_2_ nanosheet (Fig. 5d). The FE of the MoS_2_/GF cathode in CuEDTA removal reaches 29.6% at the cathodic potential of − 0.65 V, while the highest SRR is obtained at 0.042 mol cm^−2^ h^−1^. In order to demonstrate the superiority of the MoS_2_/GF-based electrochemical deposition (E.D.) system, the FE and SRR values of this system were compared with other electrooxidation-based coupling systems (Fig. 5e) [3, 9, 31, 39, 40]. The electrochemical reduction system based on MoS_2_/GF cathode is superior to the reported coupling systems based on electrooxidation, electrodeposition, photo-electrooxidation, electrocoagulation, and Fenton combined electrodeposition. Specially, the removal efficiency of the MoS_2_/GF-based electrochemical deposition system is one to three orders of magnitude higher than those of the reported systems (see details in Table S1).

### CuEDTA Removal in Zn-CuEDTA Battery

In another demonstrated system, we constructed a CuEDTA-Zn battery with MoS_2_/GF as the cathode and Zn as the anode. The galvanic cell device not only can remove the CuEDTA, but also realizes the output of electric energy. The electrode reactions involving the Zn-CuEDTA battery are shown as follows:

5 $${\text{Anode:}}\quad {\text{Zn}} - 2{\text{e}}^{ - } = {\text{Zn}}^{2 + }$$

6 $${\text{Cathode:}}\quad {\text{CuEDTA}} + 2{\text{e}}^{ - } = {\text{Cu}} + {\text{EDTA}}^{2 - }$$

**Fig. 6:**
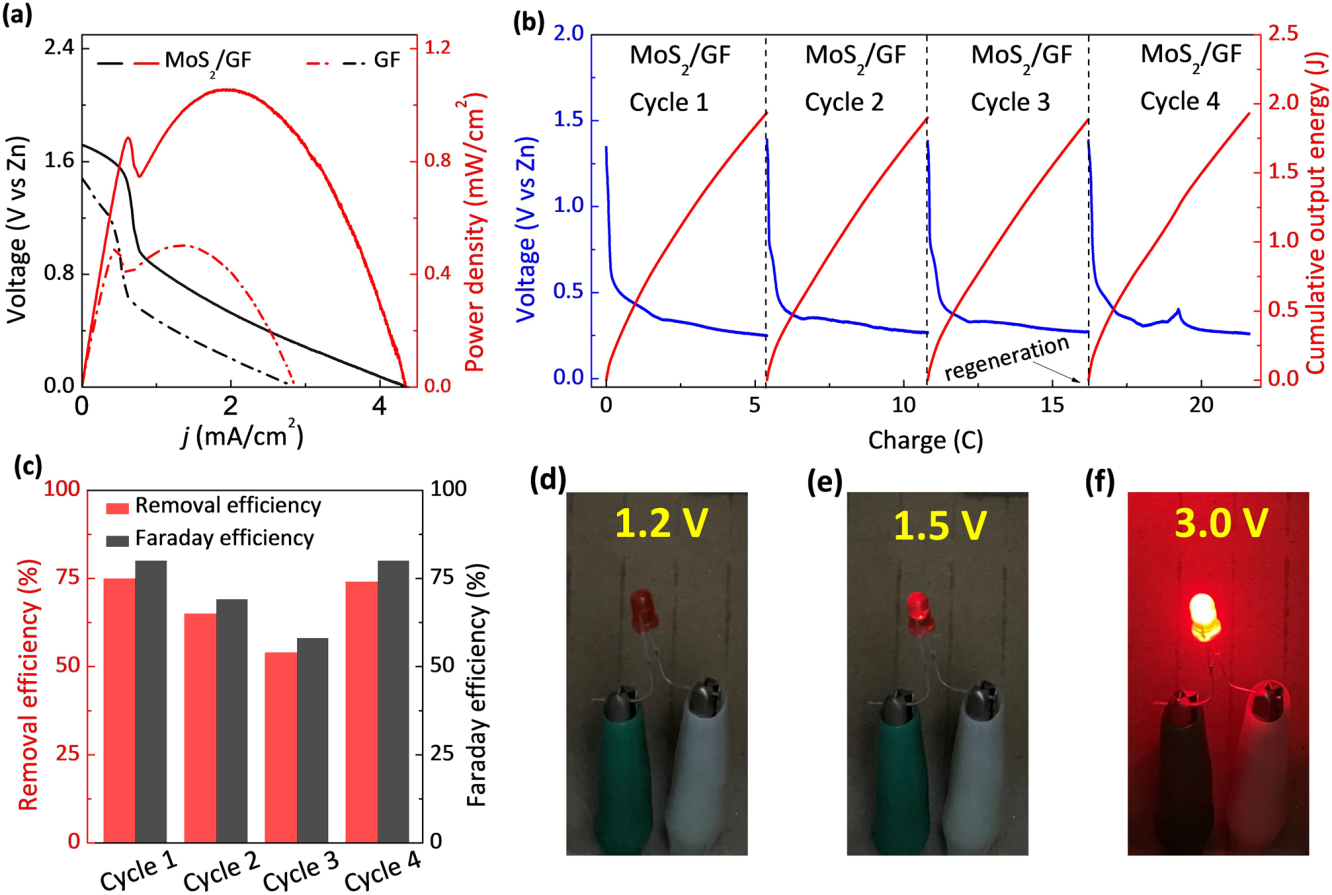
**a** Polarization curve and power density plots of MoS_2_/GF and GF-based batteries (10 mM CuEDTA). **b** Voltage and cumulative output energy at 0.5 mA cm^−2^ discharging density of MoS_2_/GF-based battery in cycling test (1 h/cycle). **c** Removal efficiency and Faraday efficiency of MoS_2_/GF-based battery. Working condition: 1 mM CuEDTA, 0.5 M Na_2_SO_4_, 30 mL solution, 3 cm^2^ electrode area. **d**–**f** 20 mW LED light powered by the CuEDTA (1 or 10 mM)||Zn (OH^−^, 1 M) battery with output potentials of 1.2, 1.5, and 3.0 V

As shown in Fig. 6a, the as-built Zn-CuEDTA battery delivers a maximum power density (10 mM CuEDTA) of 1.05 mW cm^−2^ at 1.95 mA cm^−2^ by the MoS_2_/GF electrode. In contrast, only 0.50 mW cm^−2^ of maximum power density is achieved at 1.35 mA cm^−2^ by the GF electrode. The output current density of the MoS_2_/GF electrode with even 1 mM CuEDTA is also larger than that without CuEDTA (Fig. S12), confirming that the battery performance depends on the CuEDTA reduction reaction. The output performance of our Zn-CuEDTA battery can reach the level of Zn-NO batteries, exceeding the level of some reported Zn-CO_2_ and Zn-N_2_ batteries (Table S2).

The simultaneous CuEDTA removal and output performance of MoS_2_/GF and GF electrodes at 0.5 mA cm^−2^ current density are shown in Figs. 6b and S13, respectively. The output voltage of the battery decreases gradually for both MoS_2_/GF and GF electrodes along the discharge process. This decrease can be attributed to two reasons. Firstly, the concentration polarization gradually increases due to the decrease of CuEDTA concentration (removal of CuEDTA) in the battery discharge process. Secondly, the deposition of Cu on the electrode surface leads to the electrode deactivation. By integrating the voltage-charge curve, we calculated the cumulative electrical output of the Zn-CuEDTA battery. In 1 h of discharge, the battery with the MoS_2_/GF electrode outputs 1.9 J; while the battery with the GF electrode outputs 0.6 J only.

For the battery’s cycling test, in the first three cycles, the FE and removal efficiency of the battery with MoS_2_/GF electrode gradually decrease. However, by using the same regeneration method, the FE and removal efficiency of the battery can be well recovered, as show in Fig. 6c. It is noticed that the battery with MoS_2_/GF electrode has an outstanding FE of 77%, which is several times higher than the other reported systems in CuEDTA removal (Fig. 5e). The above results indicate that the MoS_2_/GF electrode has high electrical output performance in CuEDTA-Zn battery, which is attributed to the excellent catalytic performance of MoS_2_ nanosheet in CuRR.

To demonstrate the power output characteristic of the CuEDTA-Zn battery, a 20 mW LED light was powered by the Zn-CuEDTA battery. It is found that the bulb does not emit light when the battery is constructed with 1 mM CuEDTA electrolyte (Fig. 6d). However, by increasing the concentration of CuEDTA to 10 mM, the LED light can be lit (Fig. 6e). Through putting batteries in series, the LED light emits much stronger light (Fig. 6f). The LED light experiment further confirms the power output characteristic of the Zn-CuEDTA battery and demonstrates that the higher electrical power output can be obtained by higher CuEDTA concentration in actual CuEDTA wastewater treatment. The above results show that the prepared MoS_2_/GF electrode has a practical application potential in CuEDTA-Zn battery for efficient pollutant removal and simultaneous power output.

## Conclusion

In this study, MoS_2_ nanosheet-based electrode was demonstrated in both electrolyzer and CuEDTA-Zn battery for highly efficient CuEDTA removal. The electrolyzer with MoS_2_/GF cathode has a specific removal rate up to 0.042 mol cm^−2^ h^−1^ and Faraday efficiency of 29.6%. On the other hand, the CuEDTA-Zn battery based on MoS_2_/GF electrode can simultaneously remove CuEDTA and deliver electric power output with a 1.05 mW cm^−2^ maximum energy density in 10 mM CuEDTA solution at 1.95 mA cm^−2^ current density. The battery with MoS_2_/GF cathode has a superhigh Faraday efficiency of 77% at 0.5 mA cm^−2^ discharge current density. Both systems achieve superior performances in terms of the Faraday efficiency and specific removal rate than those of the other reported electrooxidation-based systems. The density functional theory modeling confirms that the activation energy of CuRR on the MoS_2_ nanosheet surface is much lower than that of the other electrode, which explains the high activity and selectivity of MoS_2_ in CuRR. This study not only reports an efficient electrochemical reduction system for metal complex pollutant removal but also a potential electrocatalytic system for simultaneous metal complex pollutant removal and energy output.

### Supplementary Information

Below is the link to the electronic supplementary material.

Supplementary file1 (PDF 868 kb)
