# Supplementary material for: Highly Selective Electrocatalytic CuEDTA Reduction by MoS2 Nanosheets for Efficient Pollutant Removal and Simultaneous Electric Power Output
Source: Nanomicro Lett. 2023 Aug 9;15:193. doi: 10.1007/s40820-023-01166-7 (PMC10412521; doi:10.1007/s40820-023-01166-7)
Supplement: Supplementary file 1 — Supplementary file1 (PDF 868 kb) [file 40820_2023_1166_MOESM1_ESM.pdf]

Supporting Information for

Highly Selective Electrocatalytic CuEDTA Reduction by MoS_2 Nanosheets for Efficient Pollutant Removal and Simultaneous Electric Power Output

Hehe Qin[1, 2], Xinru Liu[1, 2], Xiangyun Liu[1,2], Hongying Zhao[3], and Shun Mao[1, 2, *]

[1] College of Environmental Science and Engineering, Biomedical Multidisciplinary Innovation Research Institute, Shanghai East Hospital, State Key Laboratory of Pollution Control and Resource Reuse, Tongji University, 1239 Siping Road, Shanghai 200092, P. R. China

[2] Shanghai Institute of Pollution Control and Ecological Security, Shanghai 200092, P. R. China

[3] Shanghai Key Lab of Chemical Assessment and Sustainability, School of Chemical Science and Engineering, Tongji University, 1239 Siping Road, Shanghai 200092, P. R. China

*Corresponding author. E-mail: shunmao@tongji.edu.cn (Shun Mao)

Text S1 Thermodynamics of Electrode Process

Firstly, it should be noted that the "CuEDTA" is an acronym, not the actual form of it in the aqueous solution. With different pH, there is an ionization equilibrium between $CuEDTA^{2-}$, $CuEDTAH^{-}$, and $CuEDTAH_2$ in the solution [S1]. According to the basic principles of electrochemistry, the standard equilibrium potential of the reaction ($CuEDTAH_2$ + 2e = Cu +H_2EDTA) can be calculated by the following equation:

$$\varphi = \varphi' + \frac{RT}{nF} lnK_{uns}$$

where φ' represents the standard electrode potential of redox electricity on Cu^{2+}/Cu (0.34V), R is the ideal gas constant, T is the thermodynamic temperature, n is the number of reacting electrons, F is Faraday constant, and K_{uns} is the dissociation equilibrium constant of $CuEDTAH_2$ ($10^{-18.7}$). It can be calculated that at 25 °C, the standard electrode potential of $CuEDTAH_2$ reduction is -0.212 V, which means that the standard electrode potential is lower than that of hydrogen evolution reaction (0 V). Therefore, the CuEDTA reduction is inevitably accompanied by the process of hydrogen evolution, and highly selective catalyst for CuRR is preferred to enhance the efficiency of CuEDTA reduction.

Text S2 Material Characterizations

X-ray diffraction (XRD) test was performed to characterize the crystal structure of the prepared samples on a Bruker D8 Advance X diffractometer. The size and surface morphologies of the samples were studied by scanning electron microscope (Hitachi S-

4800 SEM) with energy dispersive X−ray spectroscopy (EDS). The high-resolution transmission electron microscope images and selected area electron diffraction (SAED) patterns were obtained by transmission electron microscopy (TEM, JEM−2100) at 200 kV. The chemical composition was studied by X-ray photoelectron spectroscopy (XPS, Thermo ESCALAB 250Xi) with Mg Kα radiation source (hν = 1253.6 eV). The position of the C1s peak at 284.8 eV was employed to calibrate the position of binding energies. The phases of MoS_2 were studied by Raman spectroscopy (HORIBA HR Evolution Raman spectrometer with 532 nm laser excitation).

Text S3 Electrochemical Tests

All electrochemical measurements including CuRR and HER were conducted on a CHI 760E electrochemical workstation equipped with rotate disk electrode device in a three-electrode cell at room temperature. Pt wire and saturated calomel electrode (SCE) was used as the counter and reference electrode, respectively. The MoS_2 nanosheet electrode was prepared by dropping 5 µL of 1 mg/mL MoS_2 nanosheet dispersion on the glassy carbon electrode (diameter = 3 mm). The commercial 20% Pt/C, Cu nanoparticle, and carbon black (CB) was loaded on the glassy carbon (GC) electrode with the mass loading of 0.71 mg/cm^2. Linear scanning voltammetry (LSV) and cyclic voltammetry (CV) were measured at a scan rate of 5 mV/s and 100 mV/s in 10 mM CuEDTA and 0.5 M $NasSO_4$ solution in Ar, respectively. The electron transfer number of the reaction was calculated using the following equation:

$$j_d = 0.62nFD^{2/3}\upsilon^{-1/6}\omega^{1/2}c_0$$

where j_d is the mass transfer limit current, n is the electron transfer number, F is the Faraday constant (96485 C/mol), D is the diffusion coefficient of CuEDTA (about 7.5*10^{-6} cm^2/s), υ is the kinematic viscosity of solution (0.01 cm^2/s), ω is the rotational angular velocity, and c_0 is the reactant concentration (0.01 M). The electron transfer number of the reaction can be obtained by linear fitting of the mass transfer limiting current density at different rotational angular velocity.

Text S4 Calculation of Special Removal Efficiency, Faraday Efficiency (FE), and Cumulative Output Energy (COE)

In the current research on the removal of CuEDTA by electrochemical method, there is no clear kinetic order for the removal rate of CuEDTA. Therefore, the specific removal rate defined by us is the removal amount of CuEDTA per unit area within a certain time.

The Faraday efficiency was calculated by the following equation:

$$FE = \frac{q}{\int_0^t Idt}$$

where q, I, and t is the theoretical amount of electric charge required for CuEDTA reduction, current of the electrochemical system, and time.

The cumulative output energy in the battery was calculated by the following equation:

$$COE = \int_0^t UIdt$$

where U is the voltage recorded in chronopotentiometry method.

Text S5 Rate-limiting Step Analysis

The kinetics of the CuEDTA electroreduction process depends on the rate-limiting step. These electrode processes include reactant diffusion, pre-conversion, electron transfer ($Cu^{2+} + 2e = Cu$) and so on. Under good mass transfer conditions, pre-conversion is usually the primary rate-limiting step. Decomplexation involves the breaking of multiple chemical bonds, and the activation energy of one of these steps is typically hundred kilojoules per mole. As for the electron transfer reaction that involves the electron transfer steps, its activation energy is influenced by the overpotential:

$$\Delta G_c^{\neq} = \Delta G_c^{\neq\theta} + \alpha F \Delta\varphi$$

where $\Delta G_c^{\neq}$ and $\Delta G_c^{\neq\theta}$ is activation energy of non-standard and standard states, α is transfer coefficient, F is Faraday constant, and $\Delta\varphi$ is overpotential. The more negative the cathode overpotential, the smaller the activation energy of the cathode reaction and the faster the intrinsic rate will be. Therefore, under the reduction condition of CuEDTA ($\varphi^{\theta}(CuEDTA/Cu) = -0.21\ V$), the overpotential of the electron transfer step is in strongly polarized region ($\varphi^{\theta}(Cu^{2+}/Cu) = 0.34\ V$). The activation energy of the Cu^{2+} gaining electrons is much less than that of the decomplexation step under the condition of strong polarization. Therefore, we speculate that the rate-limiting step is decomplexation rather than electron transfer.

Text S6 DFT Calculation

The present first principle DFT calculations are performed by Vienna Ab initio Simulation Package (VASP) [S2] with the projector augmented wave (PAW) method. The exchange-functional is treated using the generalized gradient approximation (GGA) of Perdew-Burke-Emzerhof (PBE) functional. The energy cutoff for the plane wave basis expansion was set to 450 eV and the force on each atom less than 0.03 eV/Å was set for convergence criterion of geometry relaxation. Grimme's DFT-D3 methodology [S3] was used to describe the dispersion interactions. Partial occupancies of the Kohn−Sham orbitals were allowed using the Gaussian smearing method and a width of 0.05 eV. The Brillourin zone was sampled with Monkhorst mesh 3×3×1 through all the computational process. The self-consistent calculations apply a convergence energy threshold of 10^{-5} eV. A 15 Å vacuum space along the z direction was added to avoid the interaction between the two neighboring images. In the DFT calculation, because the interaction between Cu and EDTA is too strong, we added an H^+ to each step of dissociation process, so the energy of the final state is lower than that of the beginning state.

Supplementary Figures

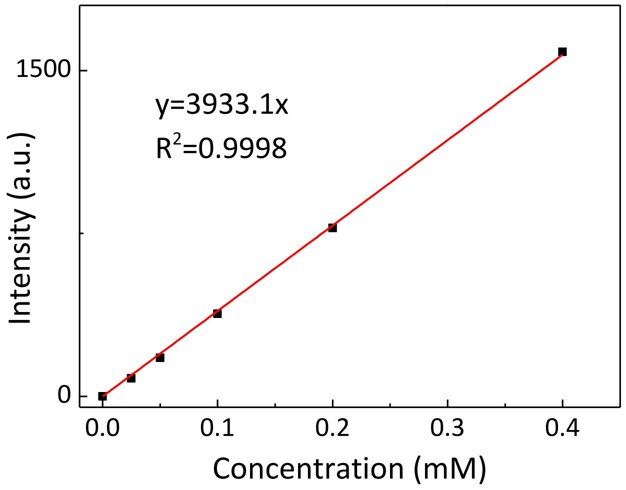

Fig. S1 The calibration curve of CuEDTA concentration determined by HPLC

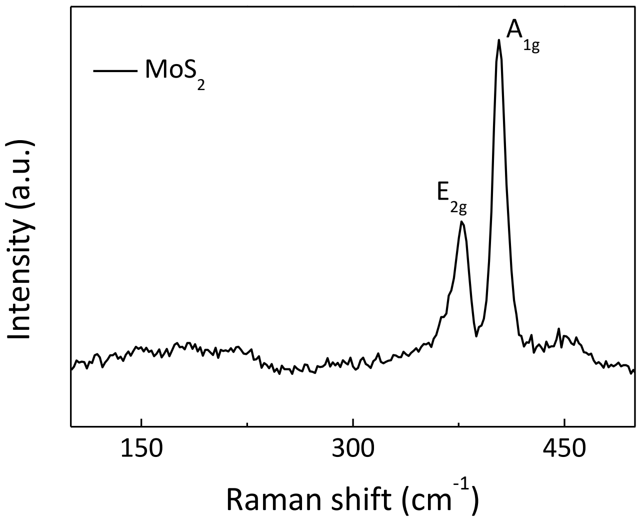

Fig. S2 Raman spectra of prepared MoS_2

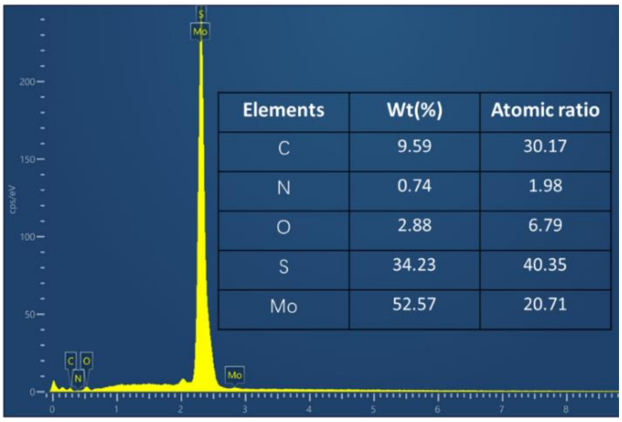

Elements	Wt(%)	Atomic ratio
C	9.59	30.17
N	0.74	1.98
O	2.88	6.79
S	34.23	40.35
Mo	52.57	20.71

Fig. S3 EDS spectra of the prepared MoS_2

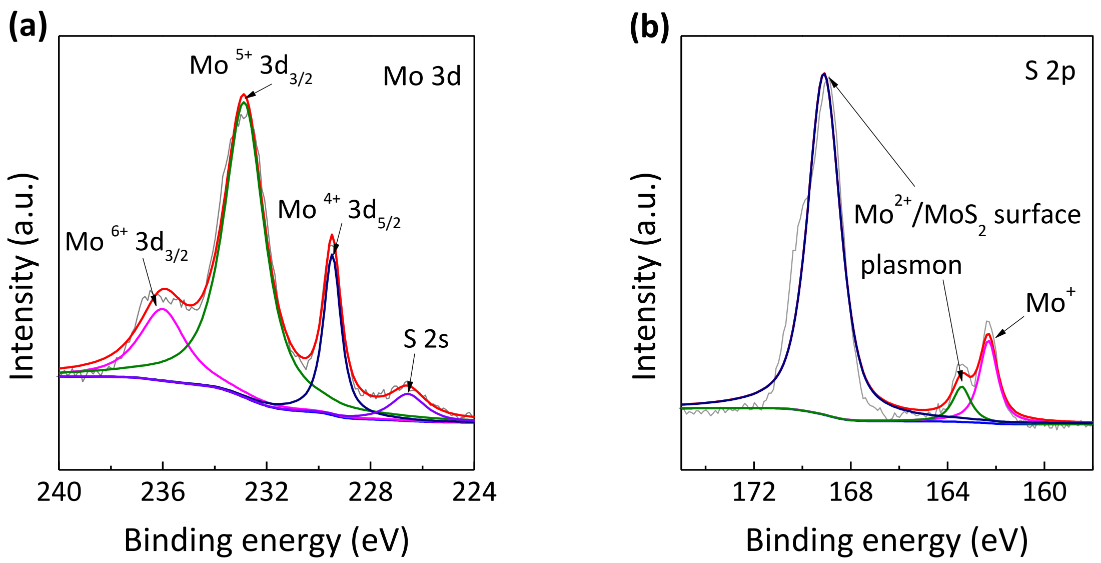

Fig. S4 Deconvoluted (Mo 3d and S 2p) XPS spectra of prepared MoS_2

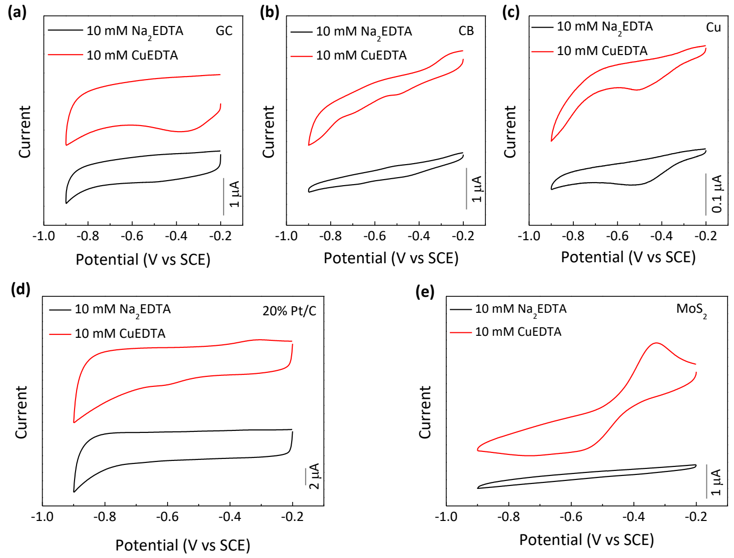

Fig. S5 CVs recorded in 10 mM Na_2EDTA (black curve) or 10 mM CuEDTA (red curve) in 0.5 M Na_2SO_4 solution at a scan rate of 100 mV/s

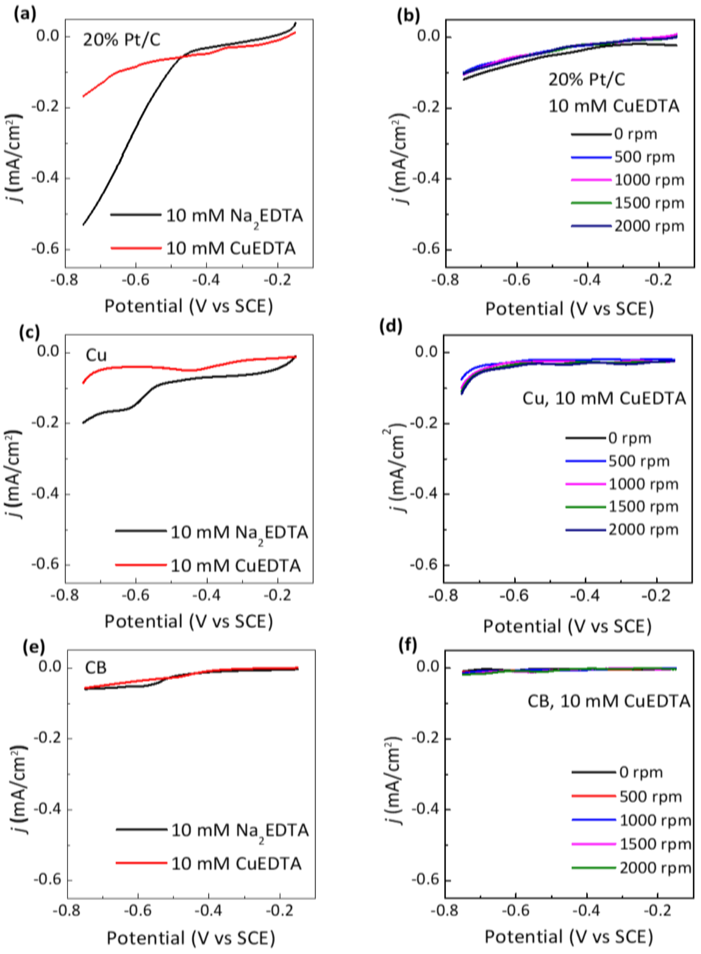

Fig. S6 Comparation of the LSVs of HER and CuRR on (**a**) 20% Pt/C, (**c**) Cu, and (**e**) CB cathodes. The rotate disk experiment of LSVs on (**b**) 20% Pt/C, (**d**) Cu, and (**f**) CB cathodes

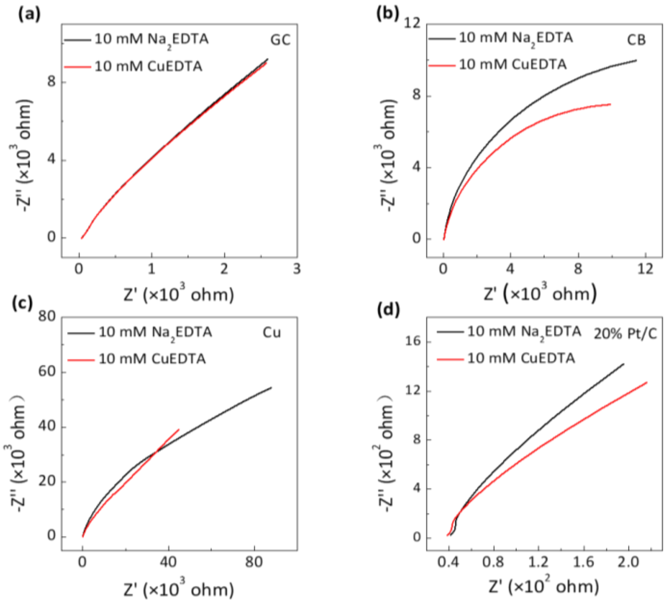

Fig. S7 Nyquist plots of the (**a**) GC, (**b**) CB, (**c**) Cu, and (**d**) 20% Pt/C cathode for CuRR and HER at -0.75 V vs SCE

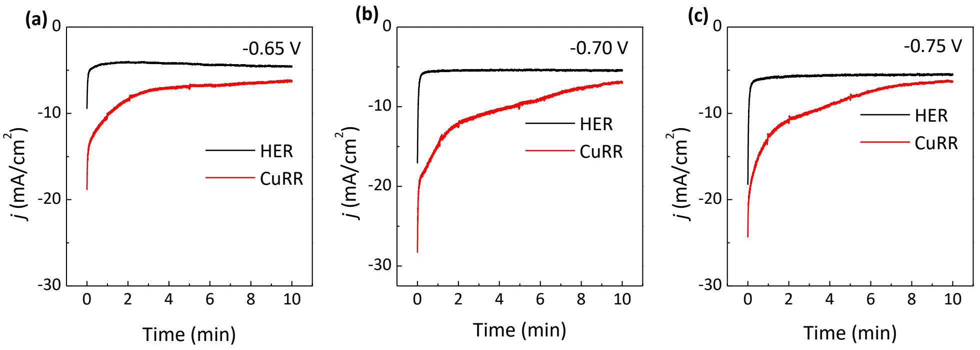

Fig. S8 Current density of HER and CuRR at different cathodic potentials on the MoS_2/GF cathode

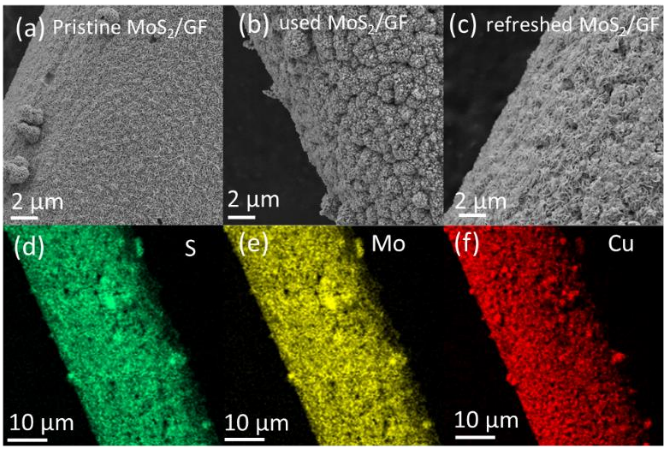

Fig. S9 SEM images of the MoS_2/GF cathode: (**a**) before the reaction, (**b**) after the reaction, and (**c**) after the regeneration. (**d-f**) The element distributions of the MoS_2/GF cathode surface after the CuEDTA removal reaction

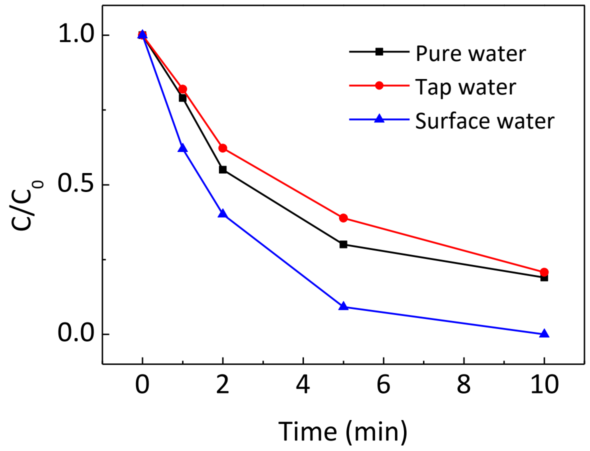

Fig. S10 CuEDTA removal efficiency in real water samples. Working condition: 1 mM CuEDTA, 0.5 M Na_2SO_4, 30 mL solution, 3 cm^2 electrode surface area

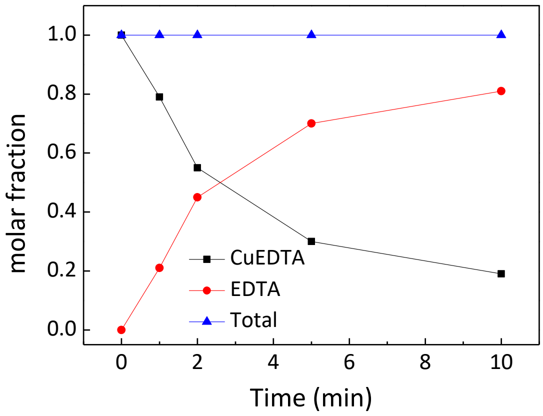

Fig. S11 The concentration evolutions of CuEDTA and EDTA during the electrochemical reduction process. Working condition: 1 mM CuEDTA, 0.5 M Na_2SO_4, 30 mL solution, 3 cm^2 electrode surface area

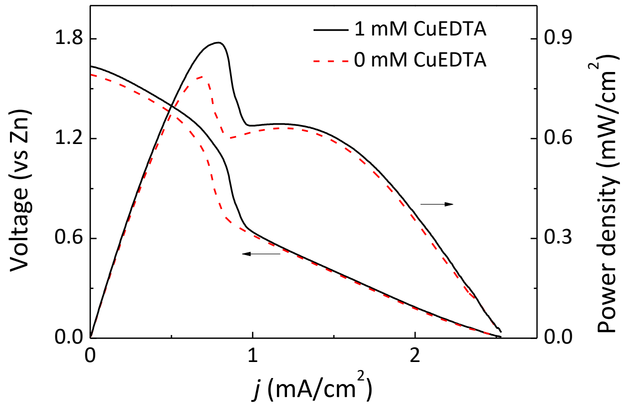

Fig. S12 Polarization curve and power density plots of MoS_2/GF with 0 mM (dash line) and 1 mM (solid line) CuEDTA

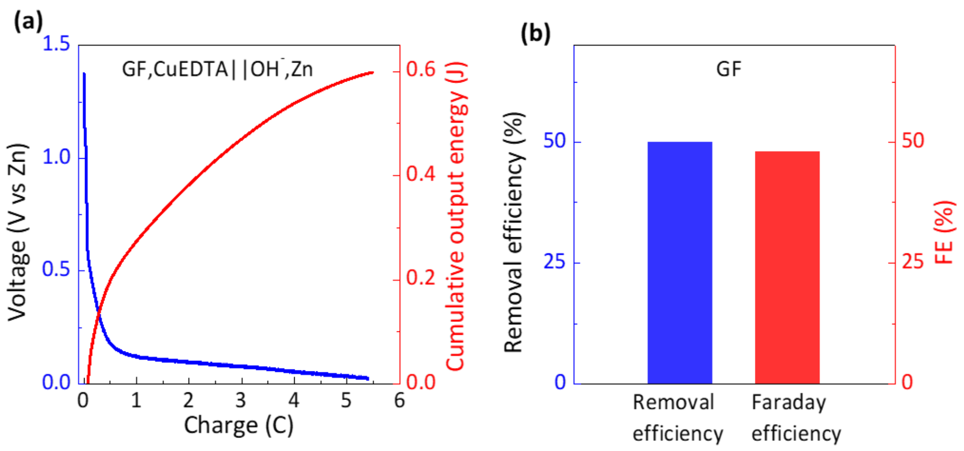

Fig. S13 (**a**)Voltage and cumulative output energy at 0.5 mA/cm^2 discharging density of the battery with GF electrode. (**b**) Removal efficiency and Faraday efficiency of GF electrode-based battery. Condition: 1 mM CuEDTA, 0.5 M Na_2SO_4, 1 h reaction time, 30 mL solution, 3 cm^2 electrode area

Table S1 Comparison of the CuEDTA removal performance by different reaction systems

Method	Electrode	Electrode area (cm^2)	Reactor volume (L)	CuEDTA Concentration (mM)	Time (min)	Current density (mA/cm^2)	SRR ($mmol/cm^2/h$)[a]	FE[a]	Refs.
E. O.+E. D.	TiO_2/Ti-S.S.	35	0.4	0.05	180	0.5	0.076	0.82%	[S4]
P. E. O.+E. D.	TiO_2/Ti-S.S.	35	0.4	0.05	180	0.5	0.14	1.49%	[S4]
P. E. O.+E. D.	TiO_2/Ti-S.S.	88.3	0.45	0.1	60	0.113	0.41	19.33%	[S5]
P. E. O.+E. D.	TiO_2/Ti-S.S.	60	0.07	0.5	120	0.2	0.23	6.25%	[S6]
E. O.+E. C.	RuO_2-IrO_2/Ti-Al	64.8	1.2	0.77	60	15.43	14.26	4.95%	[S7]
E. O.+P. E. F.+E. D	TiO_2/Ti-S.S.	88.3	0.45	0.2	60	0.2	1.02	27.32%	[S8]
E. D.	IrO_2/Ti-Pt	25	0.25	10	50	13.9	119	20.6%	[S9]
E. D.	IrO_2/Ti-Pt/Ti	25	0.25	10	50	13.9	118	20.4%	[S9]
E. D.	IrO_2/Ti-Cu	25	0.25	10	50	13.9	118	20.4%	[S9]
E. D.	IrO_2/Ti-S.S.	25	0.25	10	50	13.9	118	20.3%	[S9]
**E. D. (electrolyzer)**	**Pt-MoS_2/GF**	**3**	**0.03**	**1**	**10**	**-0.65 V**	**42.0**	**29.6%**	**This work**
**E. D. (battery)**	**Zn-MoS_2/GF**	**3**	**0.03**	**1**	**60**	**0.5**	**7.5**	**77.0%**	**This work**

[a] The EF values were calculated based on the data provided in the papers.

E.: electro

P.: photo

O.: oxidation

D.: deposition

C.: coagulation

F.: Fenton

S.S.: stainless steel

Table S2 Comparison of the output characteristics of Zn-based galvanic cell technologies

Type of battery	Peak power density (mW/cm^2)	Current density at peak power density (mA/cm^2)	Cathode	Cathodic reaction	Refs.
Zn-CO_2	0.8	1.5	CNT fiber	$CO_2 + 6H_2O + 8e^- \rightarrow CH_4 + 8OH^-$	[S10]
Zn-CO_2	0.7	2.4	Coralloid Au	$CO_2 + H_2O + 2e^- \rightarrow CO + 2OH^-$	[S11]
Zn-N_2	0.010	0.28	Cu	$N_2 + 6H_2O + 6e^-$	[S12]
Zn-N_2	0.016	0.11	VN@NSC	$\rightarrow 2NH_3 + 6OH^-$	[S13]
Zn-NO	1.04	1	MoS_2	$NO + 5H^+ + 5e^-$	[S14]
Zn-NO	1.53	2.7	NiP	$\rightarrow NH_3 + H_2O$	[S15]
**Zn-CuEDTA**	**1.05**	**1.95**	**MoS_2**	**CuEDTA+2e$^-$ → Cu+EDTA**	**This work**

CNT: carbon nanotube

VN: vanadium nitride nanodots

NSC: N, S-codoped graphitized carbon

Supplementary References

[S1] A. Eivazihollagh, J. Bäckström, M. Norgren, H. Edlund, Influences of the operational variables on electrochemical treatment of chelated Cu(II) in alkaline solutions using a membrane cell. J. Chem. Technol. Biotechnol. **92**(6), 1436-1445 (2017). https://doi.org/https://doi.org/10.1002/jctb.5141

[S2] G. Kresse, J. Furthmuller, Efficiency of ab-initio total energy calculations for metals and semiconductors using a plane-wave basis set. Comput. Mater. Sci. **6**(1), 15-50 (1996). https://doi.org/10.1016/0927-0256(96)00008-0

[S3] S.N. Steinmann, C. Corminboeuf, A system-dependent density-based dispersion correction. J. Chem. Theory Comput. **6**(7), 1990-2001 (2010). https://doi.org/10.1021/ct1001494

[S4] X. Zhao, L. Guo, B. Zhang, H. Liu, J. Qu, Photoelectrocatalytic oxidation of Cu-II-EDTA at the TiO_2 electrode and simultaneous recovery of Cu-II by electrodeposition. Environ. Sci. Technol. **47**(9), 4480-4488 (2013). https://doi.org/10.1021/es3046982

[S5] X. Zhao, L. Guo, J. Qu, Photoelectrocatalytic oxidation of Cu-EDTA complex and electrodeposition recovery of Cu in a continuous tubular photoelectrochemical reactor. Chem. Eng. J. **239**, 53-59 (2014). https://doi.org/10.1016/j.cej.2013.10.088

[S6] X. Zhao, J. Zhang, J. Qu, Photoelectrocatalytic oxidation of Cu-cyanides and Cu-EDTA at TiO_2 nanotube electrode. Electrochim. Acta **180**, 129-137 (2015). https://doi.org/10.1016/j.electacta.2015.08.103

[S7] P. Song, C. Sun, J. Wang, S. Ai, S. Dong et al., Efficient removal of Cu-EDTA complexes from wastewater by combined electrooxidation and electrocoagulation process: Performance and mechanism study. Chemosphere **287**, 131971 (2022). https://doi.org/10.1016/j.chemosphere.2021.131971

[S8] H. Zeng, S. Liu, B. Chai, D. Cao, Y. Wang et al., Enhanced photoelectrocatalytic decomplexation of Cu-EDTA and Cu recovery by persulfate activated by UV and cathodic reduction. Environ. Sci. Technol. **50**(12), 6459-6466 (2016). https://doi.org/10.1021/acs.est.6b00632

[S9] R.S. Juang, L.C. Lin, Efficiencies of electrolytic treatment of complexed metal solutions in a stirred cell having a membrane separator. J. Membr. Sci. **171**(1), 19-29 (2000). https://doi.org/10.1016/s0376-7388(99)00377-4

[S10] K. Wang, Y. Wu, X. Cao, L. Gu, J. Hu, A Zn–CO_2 flow battery generating electricity and methane. Adv. Funct. Mater. **30**(9), 1908965 (2020). https://doi.org/https://doi.org/10.1002/adfm.201908965

[S11] S. Gao, M. Jin, J. Sun, X. Liu, S. Zhang et al., Coralloid Au enables high-performance Zn–CO_2 battery and self-driven CO production. J. Mater. Chem. A **9**(37), 21024-21031 (2021). https://doi.org/10.1039/D1TA04360A

[S12] C. Du, Y. Gao, J. Wang, W. Chen, Achieving 59% faradaic efficiency of the N_2 electroreduction reaction in an aqueous Zn–N_2 battery by facilely regulating the surface mass transport on metallic copper. Chem. Commun. **55**(85), 12801-12804 (2019). https://doi.org/10.1039/C9CC05978D

[S13] X.-W. Lv, Y. Liu, Y.-S. Wang, X.-L. Liu, Z.-Y. Yuan, Encapsulating vanadium nitride nanodots into N,S-codoped graphitized carbon for synergistic electrocatalytic nitrogen reduction and aqueous Zn-N_2 battery. Appl. Catal. B: Environ. **280**, 119434 (2021). https://doi.org/https://doi.org/10.1016/j.apcatb.2020.119434

[S14] L. Zhang, J. Liang, Y. Wang, T. Mou, Y. Lin et al., High-performance electrochemical NO reduction into NH_3 by MoS_2 nanosheet. Angew. Chem. Int. Edit. **60**(48), 2110879 (2021). https://doi.org/https://doi.org/10.1002/anie.202110879

[S15] T. Mou, J. Liang, Z. Ma, L. Zhang, Y. Lin et al., High-efficiency electrohydrogenation of nitric oxide to ammonia on a Ni_2P nanoarray under ambient conditions. J. Mater. Chem. A **9**(43), 24268-24275 (2021). https://doi.org/10.1039/D1TA07455E
